# Chronopharmacology of simvastatin on hyperlipidaemia in high‐fat diet‐fed obese mice

**DOI:** 10.1111/jcmm.15709

**Published:** 2020-08-07

**Authors:** Huan Li, Anjara Rabearivony, Wenxiang Zhang, Siyu Chen, Xiaofei An, Chang Liu

**Affiliations:** ^1^ School of Life Sciences and Technology China Pharmaceutical University Nanjing China; ^2^ Department of Endocrinology Jiangsu Province Hospital of Chinese Medicine Affiliated Hospital of Nanjing University of Chinese Medicine Nanjing China

**Keywords:** chronopharmacology, hypercholesterolaemia, hyperlipidemia, simvastatin

## Abstract

The chronopharmacology refers to the utilization of physiological circadian rhythms to optimize the administration time of drugs, thus increasing their efficacy and safety, or reducing adverse effects. Simvastatin is one of the most widely prescribed drugs for the treatment of hypercholesterolaemia, hyperlipidemia and coronary artery disease. There are conflicting statements regarding the timing of simvastatin administration, and convincing experimental evidence remains unavailable. Thus, we aimed to examine whether different administration times would influence the efficacy of simvastatin. High‐fat diet‐fed mice were treated with simvastatin at zeitgeber time 1 (ZT1) or ZT13, respectively, for nine weeks. Simvastatin showed robust anti‐hypercholesterolaemia and anti‐hyperlipidemia effects on these obese mice, regardless of administration time. However, simvastatin administrated at ZT13, compared to ZT1, was more functional for decreasing serum levels of total cholesterol, triglycerides, non‐esterified free fatty acids and LDL cholesterol, as well as improving liver pathological characteristics. In terms of possible mechanisms, we found that simvastatin did not alter the expression of hepatic circadian clock gene in vivo, although it failed to change the period, phase and amplitude of oscillation patterns in Per2::Luc U2OS and Bmal1::Luc U2OS cells in vitro. In contrast, simvastatin regulated the expression of Hmgcr, Mdr1 and Slco2b1 in a circadian manner, which potentially contributed to the chronopharmacological function of the drug. Taken together, we provide solid evidence to suggest that different administration times affect the lipid‐lowering effects of simvastatin.

## 1 INTRODUCTION

In response to the autorotation of the Earth, the majority of organisms have evolved an approximate 24‐hour endogenous timing mechanism, known as the circadian clock, which controls almost all mammalian physiological and metabolic processes.^1^ It is therefore not surprising that xenobiotic metabolism, which is key for the drug efficacy, also demonstrates a robust daily oscillation.^1^, ^2^ Additionally, the target receptors, transporters and enzymes of certain drugs are rhythmically expressed in various tissues.^3^ These fluctuations contribute to circadian time‐dependent drug behaviours such as absorption, distribution, metabolism and excretion.^4^ Such rhythmicity establishes the major elements of chronopharmacology and chronopharmacodynamics.^2^, ^5^ In this sense, optimizing the administration time of the drugs during a day may increase the actions or reduce the adverse effects of drugs, benefiting patients and improving their quality of life.^5^

As one of the most widely used lipid‐lowering drugs, simvastatin (SV) inhibits enzymatic activity of hydroxymethylglutaryl coenzyme A reductase (*Hmgcr*),^6^ which is rhythmically expressed in the mouse liver.^7^ Conflicting statements exist in clinical research about the proper timing of SV administration. The authors of several randomized controlled clinical trials have reported that evening administration of SV is more efficient than morning administration.^8^, ^9^ However, Yoon and Kim recommended that morning administration is preferable.^10^, ^11^ Unfortunately, none of these studies provides a clear mechanism for explaining the chronopharmacology of SV.

To address these concerns, our study aimed to verify the chronopharmacology of SV for treating hypercholesterolaemia and hyperlipidemia in high‐fat diet (HFD)‐fed mice. We also sought to define the potential mechanisms mediating these phenomena.

## 2 MATERIALS AND METHODS

### 2.1 Experimental design

Thirty male mice were randomly divided into two groups. One group (n = 10) was fed with a normal diet (ND; Xietong biotech) whereas the other (n = 20) was fed with a HFD (60% of energy from lipid, Research Diets) for 2 months to induce hypercholesterolaemia and hyperlipidemia. Afterwards, the mice were randomly divided into six subgroups. The 10 ND‐fed mice were randomly divided into two groups, named ZT1‐ND and ZT13‐ND. Note that ZT0 (*Zeitgeber* Time 0) is the time of lights on. The 20 HFD‐fed mice were randomly divided into four groups, named ZT1‐HFD, ZT13‐HFD, ZT1‐HFD + SV and ZT13‐HFD + SV. For those three ZT1 subgroups, mice were intragastrically administrated 30 mg/kg/d of SV (CAS: 79902‐63‐9, Aladdin) or an equal volume of 0.5% sodium carboxymethylcellulose (0.5% CMC; CAS: 9004‐32‐4; Aladdin) at ZT1 for nine weeks. For those three ZT13 subgroups, mice received SV or 0.5% CMC administration at ZT13 for 9 weeks. Bodyweight, food intake and water drinking were recorded weekly. When the procedure was completed, mice were killed. The mice of ZT1‐ND, ZT1‐HFD and ZT1‐HFD + SV groups were killed at ZT1. The mice of ZT13‐ND, ZT13‐HFD and ZT13‐HFD + SV groups were killed at ZT13. The detailed methods is reported in Appendix S1.

### 2.2 Statistical analysis

All values are expressed as means ± standard deviation (SD). One‐way ANOVA was used for the data more than two groups. When the variance was homogeneous, Fisher's LSD post hoc test was conducted; otherwise, Gamos‐Howell was used. Statistical significance was set at *P* < .05. In order to compare the efficacy of SV at ZT1 and ZT13, we used the following formulas $\frac{\text{ZT}1_{\text{HFD}}‐\text{ZT}1_{{\text{HFD}}_{\text{SV}}}}{\text{ZT}1_{\text{HFD}}}*100\%$ and $\frac{\text{ZT}13_{\text{HFD}}‐\text{ZT}13_{{\text{HFD}}_{\text{SV}}}}{\text{ZT}13_{\text{HFD}}}*100\%$ for the normalization and calculate the percentage of metabolic parameters changes of ZT1‐HFD + SV and ZT13‐HFD + SV. Then, a two‐tailed Student's *t* test was used to compare the two groups.

## 3 RESULTS AND DISCUSSION

**FIGURE 1:**
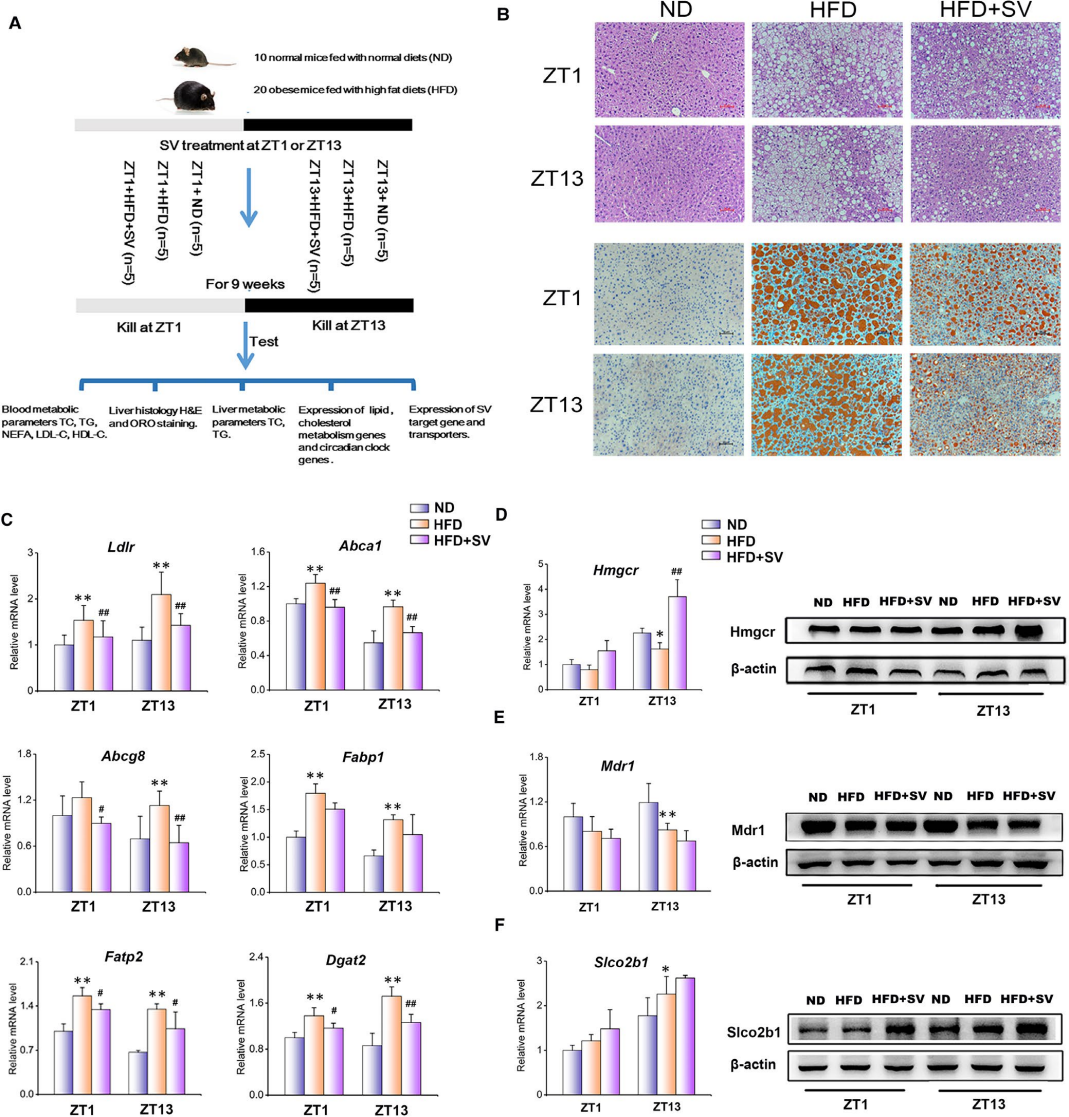
Time of simvastatin (SV dosing influences liver pathological characteristics and the expressions of lipid metabolic and drug transport genes. SV was administered to high‐fat diet (HFD) + SV groups at ZT1 or ZT13, respectively, for nine weeks, and at the same time, 0.5% CMC was administrated to normal diet (ND) and HFD groups (n = 5). A, Flow diagram of animal experiments. B, Liver H&E and ORO staining (200×). C, The mRNA expression levels of cholesterol and lipid metabolic genes. D, The expression of Hmgcr at transcriptional and translational levels. E, The expression of Mdr1 at transcriptional and translational levels. F, The expression of Slco2b1 at transcriptional and translational levels. All values are expressed as means ± SD. *represents the comparison of ND vs HFD (*P < .05, **P < .01); ^#^represents the comparison of HFD vs HFD + SV (^#^ *P* < .05, ^##^ *P* < .01)

The experimental design is illustrated in Figure 1A. After 2 months of HFD feeding, mice developed significant obesity when compared to those fed with ND. However, SV treatment decreased the weight gain in these HFD‐fed obese mice (Figure S1A). In addition, SV administration did not alter the amount of food intake and water drinking (Figure S1B,C).

We next assessed the pharmacological difference of circadian SV treatment at ZT1 and ZT13, respectively. As shown in Table 1, HFD feeding significantly increased the serum lipid levels of TC, TG, NEFA, LDL‐C and the liver injury biomarkers, such as ALT, AST and LDH, whereas the SV treatment reversed nearly all the above parameters at both examined time‐points. Consistent with these observations, liver histological and lipid examinations demonstrated that SV treatment alleviated HFD‐induced liver injury and lipid accumulation (Figure 1B). Of note, such a hypolipidaemic and hepatoprotective action of SV was more functional at ZT13 compared to that at ZT1 (Table 1). For instance, serological TC levels were decreased by 31.09% in the HFD‐fed obese mice followed by the SV treatment at ZT13, but it was only decreased by 14.30% at ZT1. For TG, the decrease was 49.11% at ZT13% vs 24.91% at ZT1.

**TABLE 1:** Changes in serum and liver metabolic parameters affected by simvastatin administration at different time‐points

Serum	ZT1			ZT13			Percentage of ZT1‐HFD + SV (%)	Percentage of ZT13‐HFD + SV (%)	*P* value
	ND	HFD	HFD + SV	ND	HFD	HFD + SV			
TC (mmol/L)	3.32 ± 0.18	7.93 ± 0.54**	6.79 ± 0.54^##^	2.93 ± 0.41	7.91 ± 0.59**	5.45 ± 0.43^##^	14.30 ± 2.62	31.09 ± 2.39	.00
TG (mmol/L)	0.95 ± 0.24	1.50 ± 0.08**	1.13 ± 0.15^##^	0.82 ± 0.26	1.57 ± 0.18**	0.80 ± 0.14^##^	24.91 ± 6.70	49.11 ± 2.92	.00
NEFA (mmol/L)	0.99 ± 0.25	1.24 ± 0.30	0.79 ± 0.13^##^	0.74 ± 0.14	0.93 ± 0.13	0.48 ± 0.11^##^	33.74 ± 14.98	48.16 ± 7.52	.12
LDL‐C (mmol/L)	0.69 ± 0.20	2.7 3 ± 0.43**	1.74 ± 0.29^#^	0.41 ± 0.06	2.50 ± 0.68*	0.89 ± 0.13^#^	36.06 ± 5.12	63.33 ± 4.79	.00
HDL‐C (mmol/L)	6.72 ± 1.76	8.17 ± 0.42	8.50 ± 0.52	6.11 ± 0.28	7.69 ± 0.71*	8.81 ± 0.31	4.01 ± 4.23	15.08 ± 7.74	.03
LDL‐C/HDL‐C	0.10 ± 0.03	0.33 ± 0.05**	0.23 ± 0.03^#^	0.07 ± 0.01	0.32 ± 0.08*	0.10 ± 0.01^#^	32.06 ± 5.41	68.06 ± 4.60	.00
ALT (U/L)	72.81 ± 29.72	227.03 ± 37.88**	132.20 ± 22.96^##^	57.37 ± 6.80	232.26 ± 64.79**	127.72 ± 36.22^##^	41.81 ± 1.91	44.79 ± 9.87	.93
AST (U/L)	116.88 ± 17.36	149.10 ± 12.08*	134.68 ± 24.74	118.75 ± 25.66	166.98 ± 16.76**	126.11 ± 18.42^#^	10.14 ± 9.62	24.61 ± 5.66	.02
LDH (U/L)	4289.63 ± 681.81	7284.26 ± 899.07**	6048.91 ± 584.95^#^	4315.51 ± 339.47	8237.80 ± 1526.14**	5668.81 ± 418.19^##^	16.73 ± 2.68	27.99 ± 3.81	.00
Liver									
TC (mmol/gprot)	0.33 ± 0.02	0.35 ± 0.01	0.32 ± 0.01	0.40 ± 0.04	0.45 ± 0.06*	0.35 ± 0.03^#^	8.21 ± 1.90	23.09 ± 3.99	.00
TG (mmol/gprot)	0.32 ± 0.03	0.60 ± 0.03**	0.49 ± 0.04^##^	0.03 ± 0.05	0.54 ± 0.06**	0.30 ± 0.04^##^	17.83 ± 3.34	43.44 ± 2.45	.00

Note

Data are listed as means ± SD.

0.00 indicates that the values are <0.005.

Abbreviation: HFD, high‐fat diet; ND, normal diet; SV, simvastatin.

* Represents the comparison of ZT1‐ND vs ZT1‐HFD or ZT13‐ND vs ZT13‐HFD (*P < .05, **P < .01).

^#^ Represents the comparison of ZT1‐HFD vs ZT1‐HFD + SV or ZT13‐HFD vs ZT13‐HFD + SV (^#^ *P* < .05, ^##^ *P* < .01).

At the molecular level, we examined the hepatic mRNA expression of key genes involved in the cholesterol and lipid metabolism regulated by SV. As shown in Figure 1C, the expression levels of *Ldlr* (uptake of exogenous cholesterol), *Abca1*/*Abcg8* (cholesterol efflux), *Fabp1*/*Fatp2* (uptake of fatty acids) and *Dgat2* (triglyceride synthesis) were increased in the liver of HFD‐fed mice, but were decreased significantly when treated with SV. Interestingly, the overall changes of these genes were more robust when observed at ZT13 compared to ZT1. Collectively, these findings suggest that SV exerts its lipid‐lowering and hepatoprotective effects in a circadian manner.

In order to determine whether the chronopharmacology of SV is mediated by the circadian clock, we measured the hepatic expression levels of key clock genes. As shown in Figure S2, whereas the HFD feeding altered the expression pattern of these clock genes, SV treatment showed a modest effect on them when compared to the corresponding HFD group. Consistently, SV failed to modulate the circadian rhythm in both *Per2::Luc* and *Bmal1::Luc* U2OS cells, including the period, amplitude and phase (Figure S3). Therefore, the chronopharmacology of SV does not appear to be directly mediated by the circadian clock either in vitro or in vivo.

To further explore the possible mechanism of the chronopharmacological effect of SV, we detected the expression of *Hmgcr*, the known target of SV.^6^ We found that SV administration was partially recovered the hepatic *Hmgcr* expression in HFD‐fed mice at both transcriptional and translational levels (Figure 1D, Figure S4A). This finding suggests that SV effectively reduced the accumulation of cholesterol, leading to the compensatory increase of *Hmgcr* expression. In addition, the drug metabolism may also play a vital role in the pharmacodynamics of SV.^12^ Given that *Mdr1*, *Mrp2* and *Slco* family members are key transporters regulating the SV efflux and uptake in the liver,^13^, ^14^, ^15^ we next checked the hepatic mRNA expression levels of these genes. As shown in Figure 1E,F and Figure S4B,C, the expression of *Mrp2*, *Slco1a4* and *Slco2b1* was induced by HFD feeding at both ZT1 and ZT13. In contrast, *Mdr1* mRNA expression was decreased by the HFD feeding. When treated with SV, the protein level of *Mdr1* was further decreased (Figure 1E, Figure S4D). Regarding the SV uptake transporters, *Slco1a4* and *Slco2b1* were expressed at a higher level at ZT13 than that at ZT1. Specifically, the hepatic protein levels of *Slco2b1* were induced by SV treatment to 3.48‐fold (ZT13) and 2.07‐fold (ZT1), respectively, when compared to their HFD‐feeding controls (Figure 1F, Figure S4E). These findings indicated that at ZT13, the expression of SV uptake transporter was induced more robustly, whereas a decrease in the expression of SV efflux transporter was observed, leading to the higher drug accumulation of SV in the liver and the better effective drug concentration.

In summary, this study demonstrated that SV administrated at both ZT1 and ZT13 produced anti‐hypercholesterolaemia and anti‐hyperlipidemia effects in HFD mice, whereas evening administration of SV was more effective. The chronopharmacological function of SV was attributed to the comprehensive action of multiple aspects: the circadian rhythm of the targeted gene *Hmgcr*, as well as the differential expression of the drug transport genes *Sloc2b1* and *Mdr1*. Moreover, we found that the chronopharmacology of SV was not directly mediated by the circadian clock. We expect that our results will be beneficial to the clinical utilization of SV and will strengthen future researchers' attention to its chronopharmacology.

## CONFLICT OF INTEREST

The authors have declared that no conflict of interest exists.

## AUTHOR CONTRIBUTIONS

**Huan Li:** Conceptualization (equal); Data curation (equal); Investigation (equal); Methodology (lead); Project administration (lead); Software (equal); Writing‐original draft (equal); Writing‐review & editing (equal). **Anjara Rabearivony:** Investigation (supporting); Methodology (supporting); Resources (supporting); Software (supporting); Writing‐review & editing (supporting). **Wenxiang Zhang:** Conceptualization (supporting); Funding acquisition (lead); Methodology (supporting); Supervision (supporting); Writing‐review & editing (supporting). **Siyu Chen:** Funding acquisition (lead); Project administration (equal); Supervision (equal); Writing‐review & editing (equal). **Xiaofei An:** Data curation (equal); Investigation (equal); Methodology (equal); Project administration (equal); Software (equal); Writing‐review & editing (equal). **Chang Liu:** Conceptualization (lead); Funding acquisition (lead); Project administration (lead); Supervision (lead); Writing‐original draft (lead); Writing‐review & editing (lead).

## Supporting information

Figures S1‐S4

Appendix S1

## Data Availability

Data supporting the findings within this article are available upon reasonable request.

## References

[1] Dallmann R , Brown SA , Gachon F . Chronopharmacology: new insights and therapeutic implications. Annu Rev Pharmacol Toxicol. 2014;54:339‐361. 24160700 10.1146/annurev-pharmtox-011613-135923 PMC3885389

[2] Zhang R , Lahens NF , Ballance HI , Hughes ME , Hogenesch JB . A circadian gene expression atlas in mammals: implications for biology and medicine. Proc Natl Acad Sci USA. 2014;111:16219‐16224. 25349387 10.1073/pnas.1408886111 PMC4234565

[3] Dallmann R , Okyar A , Levi F . Dosing‐Time Makes the Poison: Circadian Regulation and Pharmacotherapy. Trends Mol Med. 2016;22:430‐445. 27066876 10.1016/j.molmed.2016.03.004

[4] Panda S . Circadian physiology of metabolism. Science. 2016;354:1008‐1015. 27885007 10.1126/science.aah4967 PMC7261592

[5] Kalafatakis K . Rhythmicity as an important regulatory factor in complex biological systems: introduction to chronopharmacology. Ann Res Hosp. 2018;2:14.

[6] Neuvonen PJ , Backman JT , Niemi M . Pharmacokinetic comparison of the potential over‐the‐counter statins simvastatin, lovastatin, fluvastatin and pravastatin. Clin Pharmacokinet. 2008;47:463‐474. 18563955 10.2165/00003088-200847070-00003

[7] Li H , Zhang S , Zhang W , et al. Endogenous circadian time genes expressions in the liver of mice under constant darkness. BMC Genom. 2020;21:224. 10.1186/s12864-020-6639-4 PMC7066782 32160860

[8] Wallace A , Chinn D , Rubin G . Taking simvastatin in the morning compared with in the evening: randomised controlled trial. BMJ (Clinical research ed). 2003;327:788. 10.1136/bmj.327.7418.788 PMC214096 14525878

[9] Saito Y , Yoshida S , Nakaya N , Hata Y , Goto Y . Comparison between morning and evening doses of simvastatin in hyperlipidemic subjects. A double‐blind comparative study. Arterioscler Thromb. 1991;11:816‐826. 2065035 10.1161/01.atv.11.4.816

[10] Kim SH , Kim MK , Seo HS , et al. Efficacy and safety of morning versus evening dose of controlled‐release simvastatin tablets in patients with hyperlipidemia: a randomized, double‐blind, multicenter phase III trial. Clin Ther. 2013;35:1350‐1360.e1. 23998970 10.1016/j.clinthera.2013.06.020

[11] Yoon HS , Kim SH , Kim JK , et al. Comparison of effects of morning versus evening administration of ezetimibe/simvastatin on serum cholesterol in patients with primary hypercholesterolemia. Ann Pharmacother. 2011;45:841‐849. 21693699 10.1345/aph.1P511

[12] Xu C , Li CY , Kong AN . Induction of phase I, II and III drug metabolism/transport by xenobiotics. Arch Pharm Res. 2005;28:249‐268. 15832810 10.1007/BF02977789

[13] Tsamandouras N , Dickinson G , Guo Y , et al. Identification of the effect of multiple polymorphisms on the pharmacokinetics of simvastatin and simvastatin acid using a population‐modeling approach. Clin Pharmacol Ther. 2014;96:90‐100. 24598718 10.1038/clpt.2014.55

[14] Hochman JH , Pudvah N , Qiu J , et al. Interactions of human P‐glycoprotein with simvastatin, simvastatin acid, and atorvastatin. Pharm Res. 2004;21:1686‐1691. 15497697 10.1023/b:pham.0000041466.84653.8c

[15] Iusuf D , van de Steeg E , Schinkel AH . Functions of OATP1A and 1B transporters in vivo: insights from mouse models. Trends Pharmacol Sci. 2012;33:100‐108. 22130008 10.1016/j.tips.2011.10.005

