# Supplementary material for: Chronopharmacology of simvastatin on hyperlipidaemia in high‐fat diet‐fed obese mice
Source: J Cell Mol Med. 2020 Aug 7;24(18):11024–9. doi: 10.1111/jcmm.15709 (PMC7521315; doi:10.1111/jcmm.15709)
Supplement: Supplementary file 1 — Figures S1‐S4 [file JCMM-24-11024-s001.docx]

**SUPPLEMENTARY FIGURES**

**
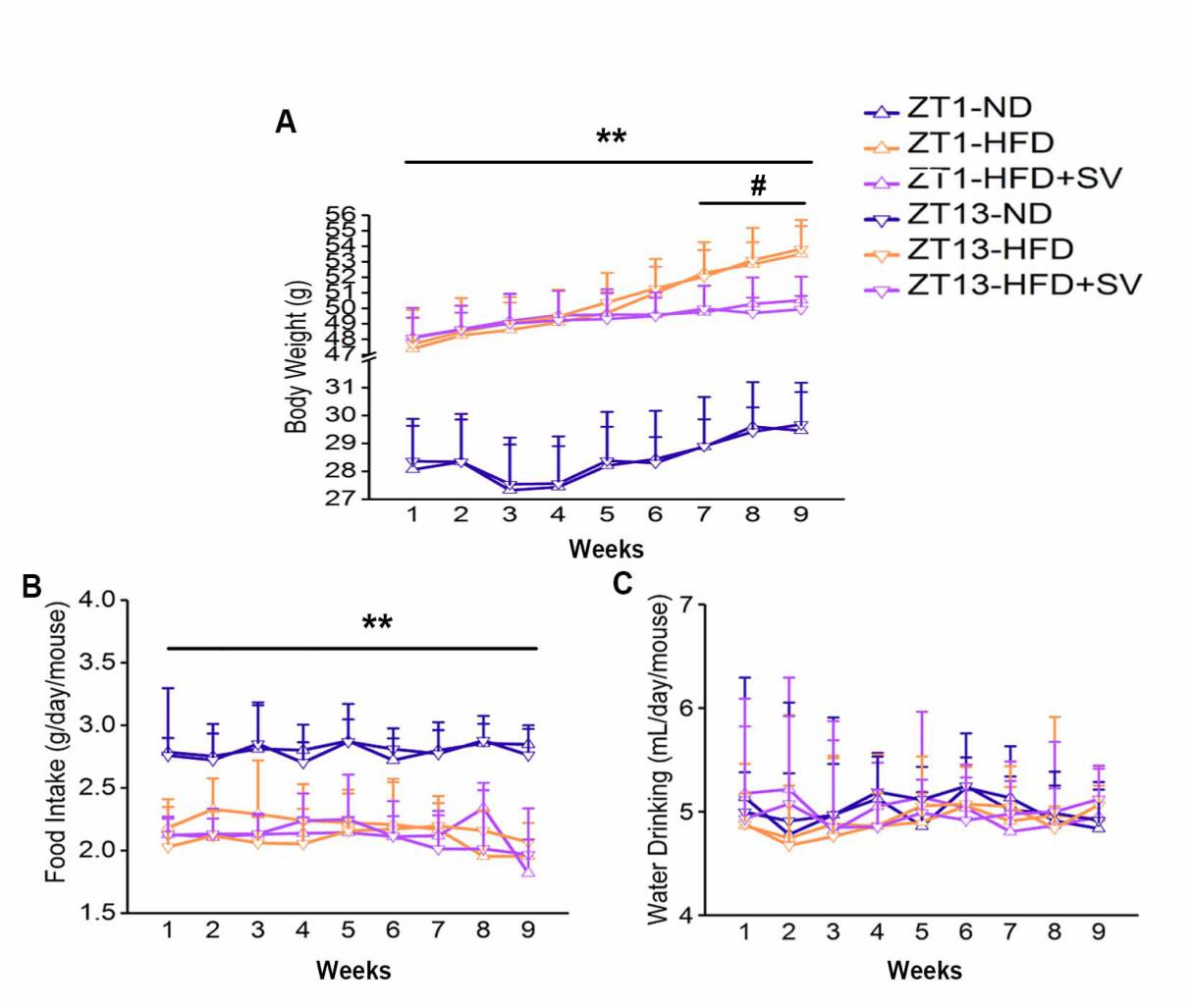
**

**Figure S1. SV treatment decreases weight gain without affecting intake of food and water.** SV was administered to HFD+SV groups at ZT1 or ZT13 respectively for nine weeks, at the same time, 0.5% CMC was administrated to ND and HFD groups (n=5). Body weight, food intake and water drinking were monitored. (A) Body Weight. (B) Food Intake. (C) Water Drinking. All values are expressed as means ± SD. ^*^ represents the comparison of ND *vs.* HFD (^*^ *P* < 0.05, ^**^ *P* < 0.01); ^#^ represents the comparison of HFD *vs.* HFD +SV (^#^ *P* < 0.05, ^##^ *P* < 0.01).

**
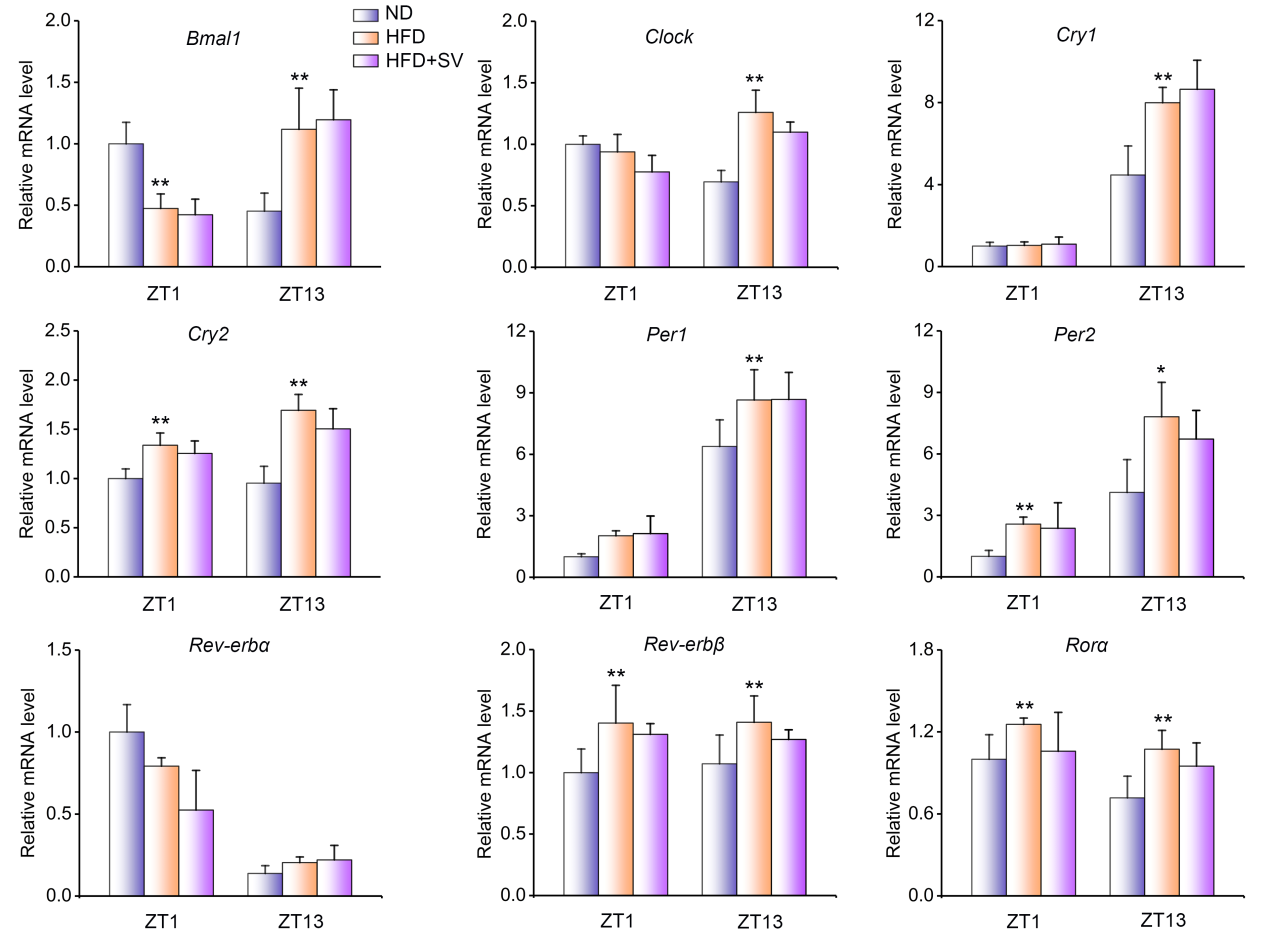
**

**Figure S2. SV has less influence in the hepatic circadian clock expression.** The RT-qPCR was conducted to check the hepatic circadian clock genes expressions for each group (n=5). All values are expressed as means ± SD. ^*^ represents the comparison of ND vs. HFD (^*^*P* < 0.05, ^**^ *P* < 0.01); ^#^ represents the comparison of HFD *vs.* HFD +SV (^#^ *P* < 0.05, ^##^ *P* < 0.01).


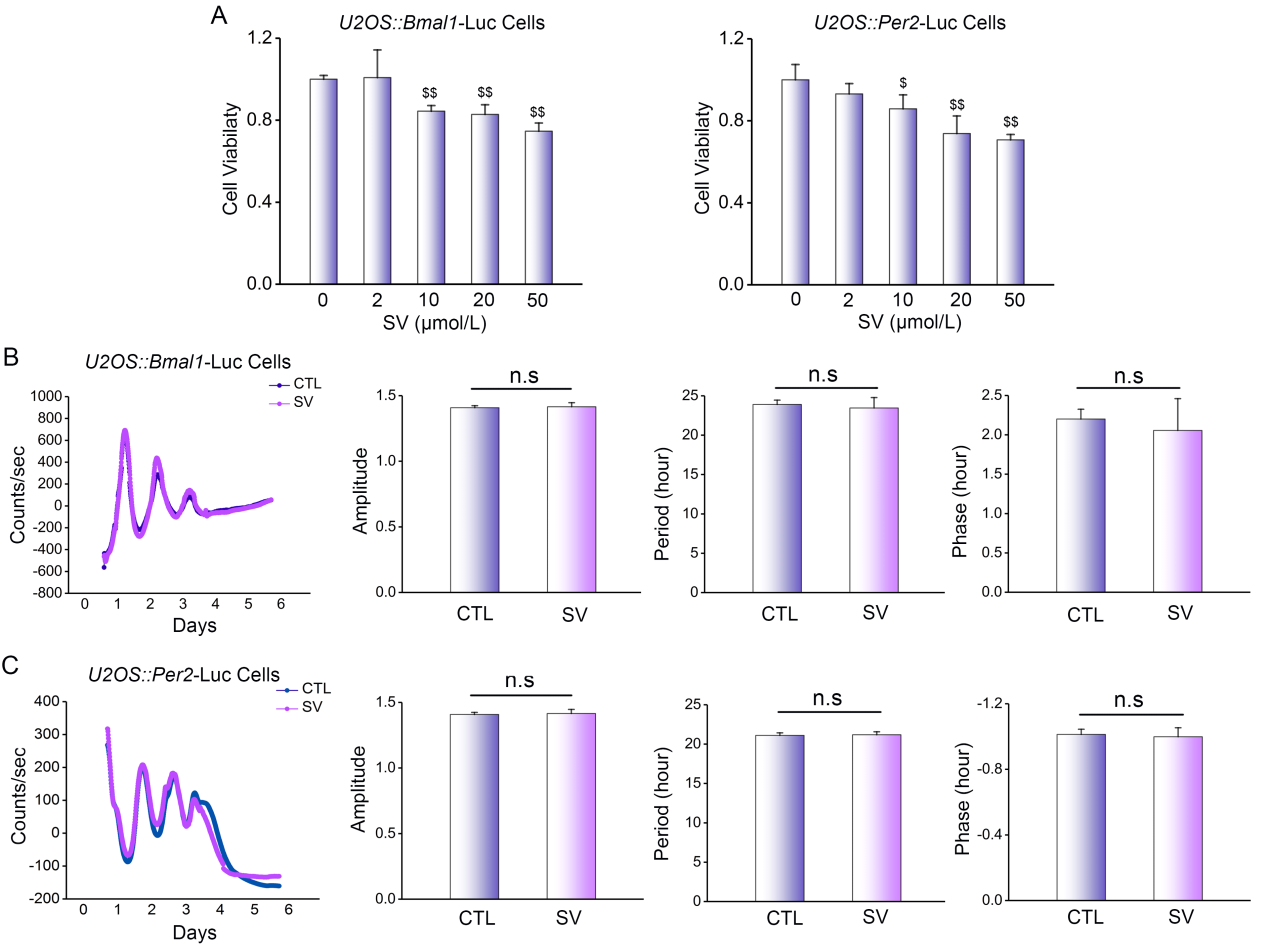


**Figure S3. SV fails to alter the oscillation of *Bmal1*::Luc and *Per2::Luc* U2OS cells.** *Bmal1::Luc* U2OS and *Per2::Luc* U2OS cells were treated with 2 µm SV or DMSO after 2-h DEX shock, then proceed to the bioluminescence experiment. (A) CCK-8 assay (n=5). (B) Real-time bioluminescence experiment of *Bmal1::Luc* U2OS cells. (C) Real-time bioluminescence experiment of *Per2::Luc* U2OS cells. All values are expressed as means ± SD. ^$^ represents the comparison of CTL *vs.* SV (^$^*P* < 0.05, ^$$^ *P* < 0.01).

**
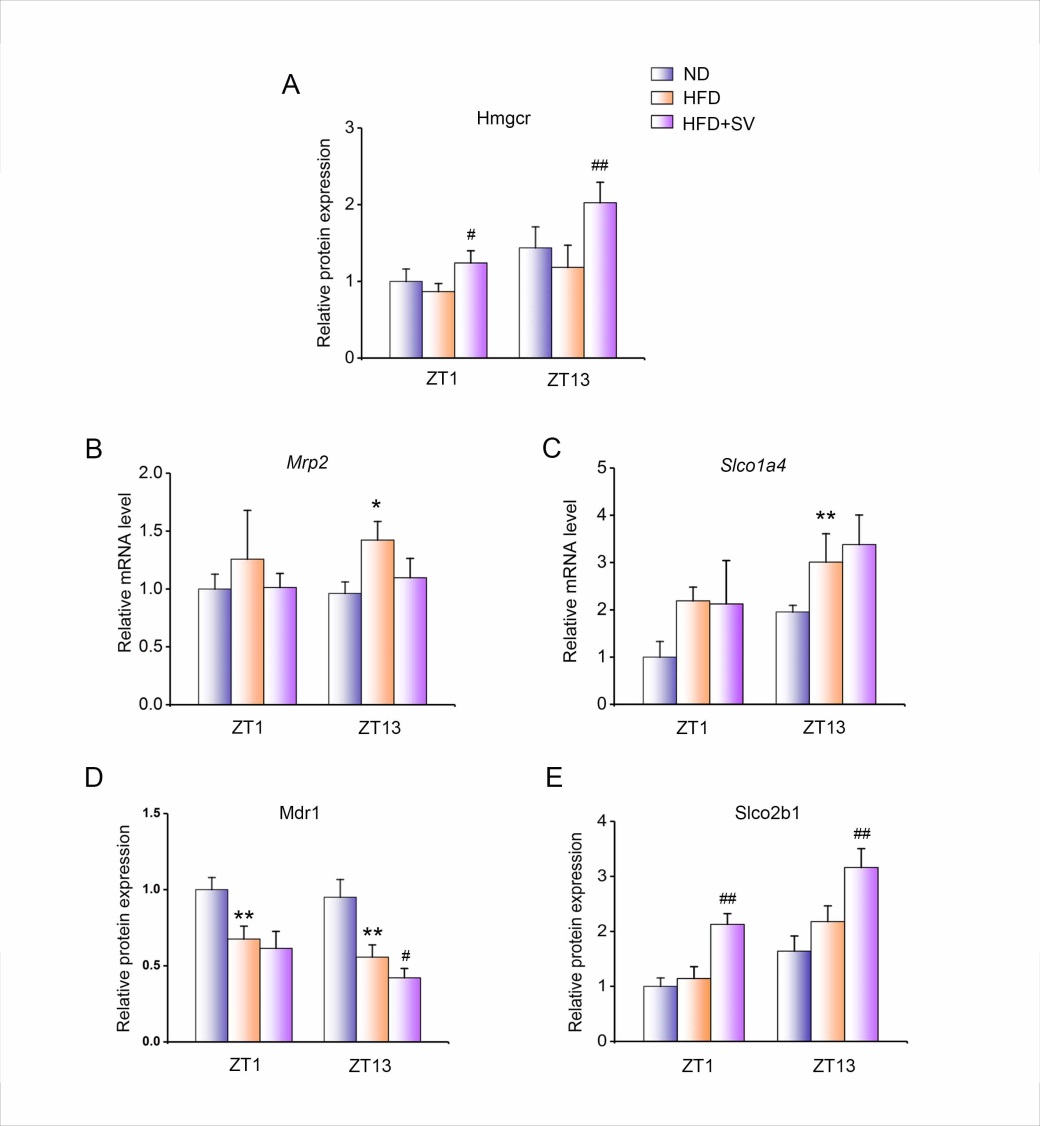
**

**Figure S4. Time of SV dosing influences expression of target and transport genes.** The mRNA expression levels of transporter genes were measured by RT-qPCR and the protein levels were quantified by western blot. (A) Statistical analysis of Hmgcr protein (n=3). (B-C) The mRNA expression levels of transporter genes (n=5). (D-E) Statistical analysis of transport proteins (n=3). All values are expressed as means ± SD. ^*^ represents the comparison of ND *vs.* HFD (^*^*P* < 0.05, ^**^ *P* < 0.01); ^#^ represents the comparison of HFD *vs.* HFD +SV (^#^ *P* < 0.05, ^##^ *P* < 0.01).
