# Supplementary material for: Chronopharmacology of simvastatin on hyperlipidaemia in high‐fat diet‐fed obese mice
Source: J Cell Mol Med. 2020 Aug 7;24(18):11024–9. doi: 10.1111/jcmm.15709 (PMC7521315; doi:10.1111/jcmm.15709)
Supplement: Supplementary file 2 — Appendix S1 [file JCMM-24-11024-s002.docx]

**Appendix S1**

**SUPPLEMENTARY MATERIALS AND METHODS**

**Animals and ethics**

Six-week-old male C57BL/6J mice were obtained from the Model Animal Research Center of Nanjing University (Nanjing, Jiangsu, China) and maintained in the animal facility under a 12h:12h light/dark cycle (light on at *Zeitgeber* time 0 (ZT0) and light off at ZT12) with free access to water and food. All animal experiments were conducted according to the Guide of the Care and Use of Laboratory Animals published by the National Institutes of Health and approved by the Laboratory Animal Care Committee at China Pharmaceutical University (Permit number SYXK-2018-0019).

**Measurement of serological parameters**

Blood was collected into non-heparinized capillary tubes and serum was routinely separated. Serum levels of alanine aminotransferase (ALT), aspartate aminotransferase (AST), lactate dehydrogenase (LDH), triglycerides (TG), total cholesterol (TC), LDL-cholesterol (LDL-C), HDL-cholesterol (HDL-C) and non-esterified free fatty acids (NEFA) were measured with commercial assay kits obtained from Jiancheng Institute of Biotechnology (Nanjing, China).

**Measurement of liver metabolic parameters**

Approximatively 100 mg of liver tissue was weighed and 900 µL ethyl alcohol was added. Electric homogenization was conducted in an iced bath to lyse the tissue. Liver homogenates were centrifuged at 3000 rpm for 10 min. Supernatants were used to quantify levels of TC and TG using commercial assay kits (Jiancheng Institute of Biotechnology, China). Protein contents of liver supernatant were measured via BCA kit (Jiancheng Institute of Biotechnology, China).

**Liver histology**

Livers were washed with ice-cold PBS and immediately fixed in 4% paraformaldehyde solution for 24 h. Specimens were then processed for paraffin embedding and tissue blocks were sectioned (4 µm thickness) for routine hematoxylin and eosin (H&E) staining. For oil red O (ORO) staining, fresh livers were frozen in liquid nitrogen, after which the frozen sections were cut into pieces of 3 μm thickness. H&E and ORO stained sections were then examined microscopically at 200 folds.

**Real-time qPCR analysis**

Total RNA was reverse transcribed into cDNA using TakaRa RT kits (Takara Bio, Japan). The 10 μL qPCR reaction mix contained 2 μL of cDNA (10 ng/ml), 5 μL of qPCR SYBR mix (Takara Bio, Japan), 0.3 μL of primer mix (25 µM each) and 2.7 μL of double distilled water (ddH_2_O). Real-time qPCR (RT-qPCR) was performed on a cycler (Roche Light Cycler 480 II), utilizing the following program: 5 min at 95 °C followed by 40 cycles of 10 s at 95 °C and 30 s at 60 °C. Obtained values were used to calculate expression of genes by the 2 ^−△△Ct^ method and normalized to the housekeeping gene *36b4*. Relative transcript levels were calculated, setting the control as 100%. Primers were designed with Primer3 software and are listed in Table S1.

**Western blotting**

Liver tissues were lysed with RIPA buffer (150 mM NaCl, 1% NP-40, 0.1% SDS, 2 mM EDTA, 50 mM Tris, pH 8.0, 1 mM PMSF and protease inhibitor cocktail). Some 30 μg of proteins were separated on 10% SDS-PAGE using electrophoresis and then transferred onto polyvinylidene difluoride (PVDF) membranes. Membranes were blocked with 5% non-fat milk and incubated overnight at 4 °C using the following antibodies: β-Actin (1:5000 dilution; Servicebio, China), Hmgcr (1:1000 dilution; Santa Cruz, USA), Slco2b1 (1:1000 dilution, Abcam, USA), Mdr1 (1:2000 dilution, Proteintech, USA).

**Clock gene oscillation by bioluminescence**

*Bmal1*::Luc and *Per2::Luc* U2OS cells (a gift from Dr. Eric Zhang, National Institute of Biological Sciences, Beijing, China) stably expressing *Bmal1*-Luc or *Per2*-Luc reporters were used to conduct luminescence recording. Each cell-line was seeded in 35 mm culture dishes at a density of 1 × 10^5^ cells/mL and incubated for approximately 24 h to reach 100% confluence with normal medium containing DMEM (Gibco, Thermo Fisher scientific, USA), 10% FBS and 1% penicillin/streptomycin. Cells were synchronized by treatment with 0.1 µM dexamethasone (DEX) for 2 h. The medium containing DEX was then replaced with working medium containing phenol-red free DMEM (Gibco), 0.1 mM D-luciferin potassium salt (Promega, Madison, WI, USA), 10% FBS, 1% penicillin/streptomycin and 10 mM HEPES. In the SV group, activated SV 2 µM was added to the working medium following the 2-h DEX shock. Following this, the dish was sealed with parafilm and placed in the LumiCycle 32 (Actimetrics, Wilmette, IL, USA) at 35°C. The SV was activated following reported methods [1,2]. SV concentration was determined by a Cell Counting Kit-8 (CCK-8) assay, following the manufacturer’s instruction (Jiancheng Institute of Biotechnology, China).

**REFERENCES**

1. Todd PA, Goa KL. Simvastatin. A review of its pharmacological properties and therapeutic potential in hypercholesterolaemia. *Drugs*. 1990; 40: 583-607.

2. Rossi J, Rouleau L, Tardif JC, Leask RL. Effect of simvastatin on Kruppel-like factor2, endothelial nitric oxide synthase and thrombomodulin expression in endothelial cells under shear stress. *Life sciences*. 2010; 87: 92-9.

**Table S1. List of Primers for RT-qPCR**

| **Primers** | **Forward ( 5'-3' sequence)** | **Reverse ( 5'-3' sequence)** |
| --- | --- | --- |
| *36B4* | GAAACTGCTGCCTCACATCCG | GCTGGCACAGTGACCTCACACG |
| *Srebp2* | GCAGCAACGGGACCATTCT | CCCCATGACTAAGTCCTTCAACT |
| *Cyp51* | GACAGGAGGCAACTTGCTTTC | GTGGACTTTTCGCTCCAGC |
| *Cyp7a1* | GGCATTTGGACACAGAAGCA | TGGAGGTTTTGCATCATGGC |
| *Ldlr* | TGACTCAGACGAACAAGGCTG | ATCTAGGCAATCTCGGTCTCC |
| *Abca1* | AACAGTTTGTGGCCCTTTTG | AGTTCCAGGCTGGGGTACTT |
| *Abcg8* | AGTTCCAGGCTGGGGTACTT | TCTCCAGGTCCTGATTGGTC |
| *Fabp1* | TACCAATTGCAGAGCCAGGA | GATGATTGATGGTTGCCGCT |
| *Dgat2* | ACGCAGTCACCCTGAAGAAC | AGGGGGCGAAACCAATATAC |
| *Bmal1* | TGGAGGGACTCCAGACATTC | TGGGACTACTTGATCCTTGG |
| *Clock* | CACTCTCACAGCCCCACTGTAC | CCCCACAAGCTACAGGAGCAGT |
| *Cry1* | AGCGCAGGTGTCGGTTATGAGC | ATAGACGCAGCGGATGGTGTCG |
| *Cry2* | TGGGCATCAACCGATGGAG | CCCATTCCTTGAACAGCCTTG |
| *Per1* | AACGGGATGTGTTTCGGGGTGC | AGGACCTCCTCTGATTCGGCAG |
| *Per2* | TGATCGAGACGCCTGTGCTCGT | CTCCACGGGTTGATGAAGCTGG |
| *Rev-erbα* | TGCAGGCTGATTCTTCACACA | AGCCCTCCAGAAGGGTAGGA |
| *Rev-erbβ* | CGCACATTGCCGATATAGGAGG | GAGACTGCCACCACCACGTACT |
| *Rora* | CCAACCGTGTCCATGGCAGAAC | GCACACAGCTGCCACATCACCT |
| *Hmgcr* | AGCTTGCCCGAATTGTATGTG | TCTGTTGTGAACCATGTGACTTC |
| *Mdr1* | ACTCGGGAGCAGAAGTTTGA | GCACCAAAGACAACAGCAGA |
| *Mrp2* | GTGTGGATTCCCTTGGGCTTT | CACAACGAACACCTGCTTGG |
| *Slco2b1* | GACTATGGCTCCAGCCTCTG | GTTGCTATGGTGGGCAAAGT |
| *Slco1a4* | GCTTTTCCAAGATCAAGGCATTT | CGTGGGGATACCGAATTGTCT |
